# Critical incidents in gynaecology: a one year audit in an academic hospital in Johannesburg, South Africa

**DOI:** 10.11604/pamj.2024.48.189.38552

**Published:** 2024-08-28

**Authors:** Christopher Chikwiri, Lawrence Chauke

**Affiliations:** 1 Department of Obstetrics and Gynaecology, University of the Witwatersrand and Charlotte Maxeke Johannesburg Academic Hospital, Johannesburg, South Africa

**Keywords:** Critical incidents, gynaecology, patient safety

## Abstract

**Introduction:**

critical incidents are among the ten leading causes of death and disability worldwide. Improving patient safety is a global priority and one way of achieving this goal is to report and analyse critical incidents. We aimed to establish the incidence, describe the profile, patient outcomes and avoidable factors associated with gynaecological critical incidents in an academic hospital in Johannesburg, South Africa.

**Methods:**

this is a retrospective descriptive analysis of critical incidents in patients admitted to gynaecology wards at Charlotte Maxeke Johannesburg Academic Hospital (CMJAH) from 1^st^ January 2019 to 31^st^ December 2019. All medical records of patients identified to have experienced critical incidents were reviewed and demographic information, timing of admission, critical incidence markers and avoidable factors were extracted and analysed.

**Results:**

there was a total of 176 critical incident events and 2082 gynaecology admissions during the 1-year study period. Only 158 critical incident files were available and complete to enable analysis. This gave a critical incidence rate of 7.6% (158/2082). The mean age (SD) of the patients was 41.1 (14.8) years and the median (IQR) duration of admission was 6 (3-10) days. The main causes of critical incidents were omission of procedures (n=45, 17.5%), deaths (n=34, 13.2%), massive transfusion (n=30, 11.7%), repeat laparotomies (n=29, 11.3%) and fistula/organ damage (n=19, 7.4%). There were 111 (70.3%) avoidable factors in the 158 critical incident cases. Most of the avoidable factors were medical care related, 53 (47.8%), followed by administrative factors, 33 (29.7%) with patient-related factors in the least at, 25 (22.5%). Critical incident forms were only filled out in 39 out of the 176 (22.2%) patients identified to have suffered a critical incident.

**Conclusion:**

the critical incidents rate in this institution is within the range reported in the literature however underreporting is a major concern. The leading causes of critical incidents were omission of procedure, followed by deaths. Approximately two-fifths of the critical incidents were associated with some form of harm, ranging from mild disability to deaths. Most of the avoidable factors were health system-related (medical care and administrative). The department should focus on improving critical incident reporting systems and the quality of care to reduce the number of critical incidents.

## Introduction

Patient safety is defined as the absence of avoidable harm to a patient during the process of health care and the reduction of risk of unnecessary harm associated with health care to an acceptable minimum [1]. Every stage during the provision of health care has a risk of a certain degree of inherent unsafety [1,2]. Critical incidents are increasingly becoming recognised as a source of harm to patients. A critical incident is defined as an event or circumstance that could have resulted or did result in unnecessary harm to a patient. Improving patient safety has become a global priority and another way to reach this goal is to report and analyse critical incidents systemically.

According to the World Health Organisation (WHO), critical incidents are among the ten leading causes of death and disability worldwide [3]. In low and middle-income countries (LMICs), hospitals experience about 134 million critical incidents each year, resulting in 2.6 million deaths annually. High-income countries estimates show that as many as 1 in 10 patients are affected by critical incidents and, approximately 50% of them are preventable [3]. Critical incidents exert a high toll on countries´ health fiscal as a consequence of additional hospital stays, medical expenses and litigation [2]. A South African study showed an increase in medical malpractice litigation in the last decade and this increase has a negative effect on public healthcare financing of public healthcare (for example, the huge legal bills could have been used to finance hiring of more staff, professional development, build hospitals and procure equipment) [4].

Literature on patient safety uses various terminology to describe critical incidents with the most common being ‘adverse clinical event’, ‘clinical incident’, ´iatrogenic events´, and ‘patient safety incident’, the latter, currently the preferred terminology. To this end, the World Alliance for Patient Safety, a section of the World Health Organization developed an International Classification for Patient Safety to devise a classification of patient safety information. Critical incidents may arise from either intended or unintended acts and are comprised of adverse events following the standard of care or near misses [5-10].

The critical incident reporting system was first published as an outgrowth of studies among military pilots in the early 1940s, by Flannagan *et al*. [11-13]. However, critical incidents in the medical field started to be considered in the 90s with the issuing of the much-cited Harvard Medical Practice Study [14,15]. This study found that about 4% of patients sustained a certain degree of harm in hospitals, and 70% and 14% of these adverse events resulted in disability and mortality respectively. Studies from developed reported that approximately 2.9%-16.6% of patients experience critical incidents and in 4.5%-20.8% of these adverse events, the patient dies. Approximately 50% of adverse events are judged to be preventable [2,16,17].

There is a lack of data and awareness regarding critical incidents in developing countries. A large-scale patient safety study in developing countries (including South Africa) showed that 8.2% of patients suffer at least one adverse event, with an average of 2.5%-18.4% per country, and, 83% of these events are judged to be preventable [9,18]. There is currently no national data on the occurrence of critical incidents in South Africa. This is an important gap especially when considering that specialty-based critical incident reporting system can give a clear picture on unit-specific safety issues which might not be necessarily exposed by hospital or national data [19]. A meta-analysis by Tanaka *et al*. found adverse event incidence of 10.8% (95%CI 9.4%-12%) among gynaecological hospital admissions. Preventability of these events in their review was 52.5% (95%CI 47.3%-57.7%) and mortality of 1.2% (95%.CL 0.25%) [17]. Lombaard *et al*. found a critical incident rate of 7.9% at Kalafong Hospital was 7.9% over a six-month period with 2.1% deaths among gynaecology admissions [20]. In another study at conducted at King Edward VIII Hospital, adverse events were observed in 11.7% of admissions, with 52% of them being avoidable [21].

We, therefore, aimed to describe and analyse critical incidents in Gynaecology Department at CJMAH. Our objectives were to describe the establish the incidence and the types of critical incidents, describe patient profile, assess outcomes and avoidable factors of patients who experienced gynaecology-related critical incidents.

## Methods

**Study design:** this research was a retrospective descriptive analysis of critical incidents in gynaecology at CMJAH from 1^st^ January 2019 to 31^st^ December 2019.

**Study setting:** this study took place in the Department of Obstetrics and Gynaecology at CMJAH, a tertiary referral hospital and one of the teaching hospitals for the University of the Witwatersrand. All patients admitted to gynaecology wards during the study period were included.

**Data collection:** the Department of Obstetrics and Gynaecology at CMJAH routinely keeps a record of patient critical incidents using a specially designed patient critical incident reporting form. This data is then captured on to the Research Electronic Data Capture (RedCap), a web-based research data management tool hosted by the University of the Witwatersrand. The incidents are discussed during the daily audit meetings as part of service improvement strategies. Furthermore, each critical incident is reported to the hospital management using a specially designed patient safety incident reporting form.

Critical incidents were sought using the following methods: 1) completed gynaecology critical incident forms; 2) analysis of Redcap database for the year 2019; 3) analysis of monthly gynaecology morbidity and mortality meeting presentations; 4) review of theatre and ward records; 5) review nursing patient safety incident reports.

We did not physically review all the files of all the admitted patients during the study period. Only medical records of patients identified to have experienced critical incidents during the study period were retrieved from the hospital filling system and demographic information, timing of admission, critical incidence marker, avoidable factors, and patient outcomes were extracted using a specially designed critical incident form and later transferred into Microsoft Excel spreadsheet to prepare for analysis.

**Data analysis:** study data was imported from Microsoft Excel for analysis. Demographic characteristics and clinical factors were analysed as follows. Categorical variables were summarised using frequencies and percentages and continuous variables using means (with standard deviations) for normally distributed data and medians (with inter-quartile range (IQR)) for non-normally distributed data. All data analyses were conducted in Stata 15.0® (StataCorp, 4905 Lakeway Drive, College Station, Texas 77845 USA).

**Ethics:** permission to access patient files was granted by the Chief Executive Officer (CEO) of CMJAH and ethics approval was obtained from the Human Research and Ethics Committee of the University of Witwatersrand (medical clearance certificate number M201083). Since this study was a retrospective audit of medical records, there was no requirement for individual patient consent. Identifying patient information was removed prior to analysis thereby always maintaining anonymity. All data was handled with the strictest confidentiality.

## Results

There was a total of 2082 gynecology admissions during the one-year study period. A total of 176 patients were recorded to have experienced critical incidents however only 158 files were analyzed (18 files and the rest were either missing or incomplete). The above translated into a critical incident rate of 7.6%. The mean age (SD) of patients was 41.1 (14.8) years, median (IQR) duration of admission was 6 (3-10) days with the longest hospital stay of 48 days. The median (IQR) parity and gravity were the same at 2 (1-3). In terms of indication/type of admissions, 60 (38%) were emergencies, elective 54 (34.2%), and 44 (27.8%) were oncology admissions. The initial intention of treatment on admission was surgical, 112 (70.9%), medical 14 (8.8%), and palliative 32 (20.3%). The most common comorbid condition was Human Immunodeficiency Virus (HIV) infection and 65 (41.1%) of the patients were HIV positive. The demographic characteristics of patients are shown in Table 1.

**Table 1:** basic demographic characteristics of patients who suffered critical incidents at Charlotte Maxeke Johannesburg Academic Hospital (CMJAH), from 1^st^ January 2019 to 31^st^ December 2019 (N=158)

Description	Mean (SD) or median (IQR) or n (%)
Mean age (SD) years	41.1 (14.8)
**Nationality**	
RSA	117 (74.1)
Other	41 (25.9)
Duration of admission (days)	6 (3-10)
**Reason for admission**	
Elective	54 (34.2)
Emergency	60 (38.0)
Oncology	44 (27.8)
**Initial treatment intention**	
Surgical	112 (70.9)
Medical	14 (8.8)
Palliative	32 (20.3)
Parity	2 (1-3)
Gravidity	2 (1-3)
HIV positive	65 (41.1)

RSA: Republic of South Africa

Overall, there were 257 adverse events in total because some patients had more than one critical incidents (Table 2). The most common category was omission of procedures, 45 (17.5%), mainly cancellations due to lack of theater time followed by deaths, 34 deaths, which was 1.63% of total admissions and contributed to 21.5% of the critical incidents. Most of the deaths, 30 (88.2%), were in the oncology population, three (8.8%) emergency admissions (one each: unsafe abortion, puerperal sepsis, pelvic inflammatory disease grade 4) and one (2.9%) elective admission (hysterectomy done for multi-fibroid uterus). A total of 30 (11.7%) patients were transfused at least four units of packed red cells. These were mostly early pregnancy complications (miscarriages and ectopic pregnancies). There were 29 (11.3%) repeat laparotomies for a number of reasons but most commonly, postoperative sepsis, and hemorrhage. Fistula and organ damages occurred in 19 (7.4%) and comprised of six ureteric injuries, five bowel injuries, four fistulae (two vesicovaginal fistulae, one rectovaginal fistula, one enterocutaneous fistula), three uterine perforations and one bladder injury. There were no anaesthetic complications.

**Table 2:** frequency of critical incidents events

Type of critical event (n=257)	n (%)
Omission of procedure	45(17.5)
Death	34 (13.2)
Transfusion > 4.0 PRC	30 (11.7)
Repeat laparotomy	29 (11.3)
Fistula and organ damage	19(7.4)
Wound sepsis	17(6.6)
ICU admission	15 (5.8)
Laparotomy for ASO	15(5.8)
Unplanned surgery	13(5.1)
Emergency hysterectomy	12 (4.7)
Organ dysfunction	9(3.5)
VTE	8 (3.1)
Delayed diagnosis	8 (3.1)
Post-op complications requiring surgery	3 (1.2)

PRC: packed red cells; ICU: intensive care unit; ASO: arterial switch operation; VTE: venous thromboembolism

Patient outcomes were assessed on a six-point scale of disability as outlined in the National Policy for Patient Safety Incident Reporting and Learning in South Africa [9]. In 50 (19.6%), critical incidents were associated with no harmful outcomes to the patient, 36 (14.0%) resulted in death, 28 (10.9%) moderate disability, 25 (9.7%) minimal disability, 14 (5.5%) permanent disability and, in five (1.9%) harm could not be specified. Overall, 103 (40.1%) out of the 257 critical incidents were associated with some form of harm to patients, ranging from mild disability to death. Table 3 is a summary of the above.

**Table 3:** avoidable factors identified in patients who experienced critical incidents

Description	n (%)
Avoidable factors in 257 critical incidents	111 (43.2)
**Patient factors**	**25(22.5)**
No antenatal care	1 (4.0)
Delay in seeking medical care	16 (64.0)
Unsafe abortion	6 (24.0)
Other	2 (8.0)
**Administrative factors**	**33 (29.7)**
Lack of health care facilities: ICU	3(9.1)
Lack of equipment and/or drugs	29 (87.9)
Lack of personnel	1 (3.0)
Transport problems	0(0.0)
Resuscitation	0(0.0)
**Medical care factors**	**53(47.8)**
Initial assessment	2 (3.7)
Diagnosis	5 (9.4)
Delay in referral	4 (7.5)
Incorrect management	3 (5.7)
Sub-standard management (correct diagnosis but wrong treatment)	2 (3.7)
Monitoring problem	1 (1.9)
Surgical complications	36 (67.9)

There was a total of 111 (43.2) avoidable factors overall among the 257 critical incidents. In a high proportion of the avoidable factors, 53 out of 111 (47.8%) were related to medical care followed by administrative factors, 33 (29.7%). The list, 25 (22%) of avoidable factors were in the category of patient-related factors (Table 4). The leading cause of avoidable factors in the medical care category was related to surgical complications, 36 (67.9%) with lack of equipment/drugs and delay in seeking medical care leading in administrative and patient-related factors at 29 (87.9%) and 16 (64.0%) respectively (Table 4).

**Table 4:** patient outcomes

Critical incident (n=257)	Number (%)
No harm	50 (19.6)
Death	36 (14.0)
Moderate disability	28 (10.9)
Minimal disability	25 (9.7)
Permanent disability	14 (5.45)
Harm not specified	5(1.95)

There was under-reporting of the critical incidents, with a reporting rate of only 22.2% during the one-year study period. The commonly reported critical incident events were operations not done due to lack of theater time in 23 of the 39 forms (59%). Most of the critical incidents were identified in the nursing theater records and ward admission books.

## Discussion

The aim of this study was to describe the rate, patient profile, outcomes, and avoidable factors associated with critical incidents in gynaecology at the study site during the one-year study period. The study found a critical incidence of 7.6% from all gynecological hospital admissions during the study period. This result is slightly lower than the WHO estimate of 1 in 10 patients [3] and that of a meta-analysis by Tanaka *et al*. of 10.8% [17]. Furthermore, our results are like a study by Lombaard *et al*. at Kalafong Hospital, Pretoria but higher when compared to a study by Matsaseng *et al*. at King Edward VIII Hospital, Durban. The two South African studies reported a critical incident rate of 7.9% and 11.7% respectively [20,21]. The Kalafong study covered a period of six months and the Durban study, nine months. The mean age (SD) of patients who had critical incidents in this study was 41.1 (14.8) years with a median gravidity and parity of two. Increasing age is an established risk factor for critical incidents [17,20]. Similarly, Wilson *et al*. showed that critical incidents in developing countries increase with patient´s age [18]. This study also showed that the rate of critical incidents increases with length of hospital stay (median length of hospital stay ranged from 2 to 7 days), which is also comparable with our results [18]. Our study could not draw an inference on the association between patient demographic characteristics and critical incidents rate because we did not analyse data of all the admitted patients. What was evident though was that critical incidents resulted in increased hospital stay. The longest hospital stay was 48 days, for a patient who was electively admitted for a diagnostic laparoscopy for Mullerian agenesis which was complicated by abdominal sepsis, leading to three relook laparotomies. Another example is a patient who was admitted three times with a multifibroid uterus for an elective hysterectomy. The procedure was cancelled three times due to a lack of theatre time, despite spending a total of 17 days in the hospital. The above example illustrates how critical incidents negatively affect public healthcare financing through the extra cost of prolonged hospital stay [4]. Patients are also affected through loss of income, disability, and medical expenses not forgetting the psychological and social impact.

Our most common type of critical incidents was omission of procedure (n=45, 17.5%), which was mainly cancellation of surgical operations for various reasons. A total of 26 operations were cancelled due to lack of theatre time, while other reasons for cancellation were lack of intensive care unit beds, staff, and linen shortages, and inadequate preoperative work-up and preparation. We could not establish the impact of this omission on patient outcomes. It has been reported that routine incident reporting systems are poor at identifying patient safety incidents that result in patient harm [11]. This contrasts with findings by Matsaseng *et al*. [21] where therapeutic mishaps were the most common causes of gynecological critical incidents. The impact of the omission of procedure on patient outcomes was not assessed however 40.1% of the 257 critical incidents were associated with some harm to the patient, ranging from mild disability to death. Furthermore, omission of exert a high toll on the financial status of the hospital and patients because of additional hospital stay, medical expenses, and a loss of income. It may also result in the loss of confidence and patient dissatisfaction with public healthcare services.

There were 34 deaths (1.6% of 2082 admissions and 13.2% of the 257 critical incidents). A meta-analysis on gynaecological hospital admissions found a low mortality rate of 1.2% (95% CI 0 -2.5%) [17]. Similarly, the two South African studies on gynaecological critical incidents discussed previously reported a mortality of 2.1% [20,21]. Thirty of the deaths in this study were in the oncology/palliative admissions group in which patients had advanced disease and death was mostly unavoidable. Only four deaths were outside the oncology population (one each: unsafe abortion, puerperal sepsis, PID grade 4, and post hysterectomy for multifibroid uterus). These four deaths where avoidable therefore emphasis should be put in auditing non-oncology deaths to reduce avoidable critical incidents and related deaths [15,17]. The mortality rate in this study would have been much lower (0.2% of total admissions) if gynaeoncology-related deaths were excluded. Whilst all deaths are reportable events, there is a need to refine our definition of deaths as a critical incident marker. Only deaths that are directly caused or brought forward in the short term during the process of health care should be recorded as critical incidents as the aim is to reduce such deaths.

The critical incident events that resulted in most moderate to severe disability were fistula and organ damages at surgery (n=19, 74%). There were six cases of ureteric injuries, five cases of bowel injuries, four fistulae formation, three uterine perforations, and one bladder injury. There were six laparoscopic surgery complications resulting in unplanned laparotomy. Of the six, two were because of abdominal wall haematoma, another two were due to post-operation hemorrhage following a laparoscopically assisted vaginal hysterectomy, one uterine perforation by a uterine manipulator, and one, for lost instrument. Our research did not look at complication rates of different gynecological operations to compare with local and international literature, we therefore recommend that future studies look at this aspect. Two of the injuries occurred during radical hysterectomy in patients with cervical cancer, one during a hysterectomy for a multifibroid uterus, and unexpectedly three ureteric injuries from oophorectomy for ovarian masses. The incidence of ureteric injury reported in this study is higher compared to the study by Matsaseng *et al*. (zero in 1866 admissions, 9-month study period) and Lombaard *et al*. (one in 1165 admissions, 6-month study period) [20,21]. The higher numbers of laparoscopy-related ureteric injuries in this study might be pointing toward inadequate laparoscopy surgical skills and the need for training in this area.

While we report on critical incidents in gynecology, it must be noted that there is currently a lack of universally accepted criteria to assess patient harm. This lack of uniformity in data made it challenged when researchers wish to compare critical incident patient outcomes with between different settings and studies. We used the classification as described in the National Policy for Patient Safety Incident Reporting and Learning in the Public Health Sector of South Africa, July, 2016 [9]. The other challenge was that there was no long-term follow-up of patients to determine the duration of harm, additional intervention needed or pick up harm which was not apparent on the initial assessment. Other files were incomplete; hence harm could not be specified. Brennan *et al*. stated that critical incidents will not always point to substandard care nor do their absence point to good quality care [15]. The occurrence of unavoidable critical incidents is an inherent risk and part of medical care. However, the goal of studying critical incidents is to lower the incidence of avoidable factors thereby improving patient safety [17]. There were avoidable factors in 43.2 avoidable factors in patients who suffered critical incidents in our study. This is lower than the 50% reported in other studies [2,3,16,17]. Most of the avoidable factors were health system-related (medical care and administrative factors). Improving patient safety at a gynaecology department administrative level can be challenging as it involves complex issues which involve clinical care and administrative factors some of which are outside the control of clinicians. These include increasing patient population size without a corresponding increase in hospital beds or staff, and limited healthcare budgets, all of which need the political will to improve. We still saw a worrying number of unsafe abortions, (n=6, 5.4%), contributing to avoidable critical incidents events. This is despite the provisions of the Choice on Termination of Pregnancy Act which strives to provide for safe abortions. An equally worrying result is the number of patients, n=16 (14.4% of avoidable factors), who delay seeking medical care. This is important in oncology where delay could result in the patient presenting with advanced disease. More community health education is needed to improve early health-seeking behaviour and awareness on the availability of safe abortion services.

We audited the critical incident reporting system in our clinical department. Of the 158 patients who suffered critical incidents, only 39 patients (22.2%), had critical incident forms filled in. Despite the advantages of critical incident reporting, it is known that it is an underutilised tool in health care because of widespread underreporting found in different studies [12,14]. Mahajan *et al*. noted that underreporting especially by doctors remains a significant problem [12]. This observation is like our study as most of the critical incidents were not reported by doctors but were found in nursing records (theatre and ward admission books). For example, all six cases of ureteric injuries found in our study were not reported by doctors but were found in nursing theatre records. Factors responsible for poor reporting include fear of punitive actions, discrimination at the workplace, and litigation [12]. Other factors for underreporting are incidents not recognized and no clarity on what should be reported. No anaesthetic complications were reported in our research. It is however more likely to be part of the underreporting than a real absence of anaesthetic critical incident events. Similarly, our electronic critical incident reporting system, done on RedCap daily surveys, also recorded some critical incidents. However, the data is of limited value, as the system doesn´t specify the type of critical incident event, nor does it capture any details of the patients involved. We therefore recommend modification of the electronic system so that it can capture data which is useful to analyse critical incidents.

**Limitations:** this was a retrospective descriptive study and we faced limitations inherent to such studies such as missing patient files and data variables incompletely filled in. Our study may also have been affected by observer bias when it comes to analyzing patient outcomes. We relied only on critical incidents which were reported in various records, and we did not go through all the medical records of patients admitted during the study period to find unreported events.

## Conclusion

The critical incidents rate in this institution is within the range reported in the literature however underreporting is a major concern. The leading causes of critical incidents were omission of procedure, followed by deaths. Approximately two-fifths of the critical incidents were associated with some form of harm, ranging from mild disability to death. Most of the avoidable factors were health system-related (medical care and administrative). The department should focus on improving critical incident reporting systems and the quality of care to reduce the number of critical incidents.

### What is known about this topic

- Critical incidents are among the 10 leading causes of death and disability worldwide;
- There is limited research on patient safety despite critical incidents causing more deaths than AIDS, motor vehicle accidents, or breast cancer;
- The critical incidents reporting system is an underutilized tool for improving patient safety.

### What this study adds

- Our research adds data about the incidence of specialty-based critical incidents in low-income settings;
- Identified the leading causes of critical incidents as well as avoidable factors; this information can be used to improve the quality of care, reduce patient harm, and improve patient outcomes;
- Highlight need for precise definitions of critical incidents terms and need for standardized critical incidents reporting systems.
